# Role of Local Evidence in Transferring Evidence-Based Interventions to Low- and Middle-Income Country Settings: Application to Global Cancer Prevention and Control

**DOI:** 10.1200/GO.22.00054

**Published:** 2022-08-12

**Authors:** Mark Parascandola, Gila Neta, Ramzi G. Salloum, Donna Shelley, Anne F. Rositch

**Affiliations:** 1 Center for Global Health, National Cancer Institute, Bethesda, MD; 2 Division of Cancer Control and Population Sciences, National Cancer Institute, Bethesda, MD; 3 Department of Health Outcomes & Biomedical Informatics, University of Florida College of Medicine, Gainesville, FL; 4 Department of Policy and Public Health Management, NYU School of Global Public Health, New York, NY; 5 Department of Epidemiology, Johns Hopkins Bloomberg School of Public Health, Baltimore, MD

## Abstract

Although the global burden of cancer falls increasingly on low- and middle-income countries (LMICs), much of the evidence for cancer prevention and control comes from high-income countries and may not be directly applicable to LMIC settings. In this paper, we focus on the following question: When the majority of the evidence supporting an evidence-based intervention or implementation strategy comes from high-income countries, what local, contextual evidence is needed when transferring and adapting an intervention or strategy to a specific LMIC setting?

**METHODS:**

We draw on an existing framework (the Population, Intervention, Environment, Transfer-T process model) for assessing transferability of interventions between distinct settings and apply the model to two case studies as learning examples involving implementation of tobacco use treatment guidelines and self sampling for human papillomavirus DNA in cervical cancer screening.

**RESULTS:**

These two case studies illustrate how researchers, policymakers, practitioners, and consumers may approach the need for local evidence from different perspectives and with different priorities. As uses and expectations around local evidence may be different for different groups, aligning these priorities through multistakeholder engagement in which all parties participate in defining the questions and cocreating the solutions is critical, along with promoting standardized reporting of contextual factors.

**CONCLUSION:**

Local, contextual evidence can be important for both researchers and practitioners, and its absence may hinder translation of research and implementation efforts across different settings. However, it is essential for researchers, practitioners, and other stakeholders to be able to clearly articulate the type of data needed and why it is important. In particular, where resources are limited, evidence generation should be prioritized to address real needs and gaps in knowledge.

## INTRODUCTION

Substantial evidence exists to support the effectiveness of several cancer control interventions and implementation strategies. The US National Cancer Institute's Evidence Based Cancer Control Programs database, for example, includes more than 200 entries.^1^ Moreover, effective implementation of cancer control interventions, including policies, programs, and guidelines, has led to a substantial decrease in cancer mortality in the United States and other high-income countries (HICs).^2^

**CONTEXT**

**Key Objective**
When a cancer control intervention or strategy is transferred from one context to another, particularly when moving from a high-income to a low- or middle-income setting, questions are often raised about the need for local evidence. We draw on the Population, Intervention, Environment, Transfer-T process model for assessing transferability of interventions to understand contextual data needs.
**Knowledge Generated**
Two case studies involving tobacco control and cervical cancer prevention illustrate how researchers and practitioners may approach the need for local evidence from different perspectives and with different priorities. Multistakeholder engagement, in which all parties participate in defining the questions and cocreating the solutions, is critical to ensure that relevant data needs are addressed.
**Relevance**
Through researchers and practitioners working together from the start to identify data gaps relative to the local context, evidence generation can be focused where it is most critical to support implementation of evidence-based interventions across diverse contexts.

Although cancer incidence rates have been falling in HICs, they have been rising in many low- and middle-income countries (LMICs), and this disparity is projected to increase.^3^ Effective implementation of evidence-based cancer control measures in LMICs is critical to addressing the global cancer burden. However, much of the evidence for cancer prevention and control comes from HICs and may not be directly applicable to LMICs because of competing health priorities, limited resources, health system structure and capacity, and other cultural or sociopolitical differences. Cancer control interventions and implementation strategies developed in HICs may require adaptation for successful implementation in LMICs.^4,5^ For example, human papillomavirus (HPV) vaccinations have been scaled up across many HICs, but implementation lags in LMICs. In response, the WHO launched a global initiative in 2020 that acknowledged the need to develop context-specific strategies to accelerate the elimination of cervical cancer across diverse settings.^6^ Additionally, breast cancer, now the most frequently occurring cancer worldwide, is increasing in many LMICs. However, large-scale mammography programs used in many HICs may not be appropriate in some settings because of cost and limited health care infrastructure; thus, resource-constrained settings may require greater reliance on other strategies such as community-level awareness and training of frontline health workers to promote early detection.^7^

When transferring an intervention or implementation strategy (eg, training program or system changes) from a HIC to an LMIC setting, questions are often raised about the need for local evidence. Will the intervention be effective in this setting? Will it be accepted by the community? What adaptations are needed? The successful translation of cancer control interventions and practices in LMICs is a function not only of the effectiveness of the intervention, but also of how the intervention fits within the environment in which it is applied.^8^ Thus, it is important to understand factors at the individual, community, and systems level that may influence how an intervention is delivered and adopted in a novel setting. At the same time, however, a narrow focus on the local context may lead to excessive reliance on small pilot studies (termed pilotitis), which may hinder or slow efforts to bring interventions to scale.^9^ Thus, striking the right balance of responding to the local context and not undertaking overly burdensome or unnecessary research or data collections tasks is of critical importance to accelerating cancer control in LMICs.

In this paper, we focus on the following question: When the majority of the evidence supporting an evidence-based intervention (EBI) or implementation strategy comes from HICs, what local, contextual evidence is needed when transferring and adapting an intervention or strategy to a specific LMIC setting? What factors should researchers, practitioners, and decision makers consider when weighing the need for local evidence? We review key concepts of transferability of interventions and use two case studies in cancer control to demonstrate how these concepts can help to identify when local, context-relevant evidence is needed. We draw on an existing framework (the Population, Intervention, Environment, Transfer [PIET]-T process model) for assessing transferability of interventions between distinct settings and apply the model to two case studies. This exercise yielded novel lessons for considering local data needs for transferring an intervention or strategy to a new setting. We conclude by suggesting best practices for researchers engaged in adapting and testing cancer control interventions in LMIC settings.

## LOCAL EVIDENCE AND TRANSFERABILITY OF INTERVENTIONS

Local evidence has been defined as evidence that is available from the specific setting(s) in which a decision or action on a policy or program option will be taken. ^10^ We contrast local evidence with global evidence, which is generalizable but may not take into account characteristics or important nuances of a specific setting. There are a variety of situations where local data may be expected or required, particularly when the target setting population and environmental context differs significantly. For example, local evidence may be needed when there are no equivalent data available for a particular cultural or health system context. Local evidence may also be required to guide development or testing of adaptations to existing interventions and implementation strategies. Beyond translation, there may be differences in culture or climate^11^ in the way people communicate risk that influence modifications in how screening and prevention programs are promoted and implemented. For example, although the addition of graphic warning labels on cigarette packages is likely to affect smokers in a similar way across countries and regions, the specific content of those labels should be tailored to the local cultural and legal context. In addition, local evidence can be essential to estimate cost,^12^ acceptability, and feasibility in a given context, and local evidence may be required in response to demands from policymakers as a condition for action, decision making, or prioritization. In practice, however, such needs for local data are sometimes made with little explanation of or justification for what kind of data or evidence is needed or without a definition of what is meant by local evidence. Additionally, stringent expectations for local evidence may lead to discounting of evidence-based public health interventions, as has occurred around decisions to adopt tobacco control policies or incorporate HPV self-sampling into cervical cancer prevention programs.^13,14^ Thus, substantive ongoing engagement with all systems stakeholders is essential to generate research questions and answers that are relevant to transferring an intervention to an LMIC.^15,16^

Implementation science offers methods and frameworks for evaluating factors influencing implementation of EBIs at multiple levels and identifying and tailoring implementation strategies to address barriers across varying settings. The concept of transferability is essential here, as researchers and stakeholders consider the spectrum of evidence needed to take an intervention from one setting to another and assess the extent to which outcomes observed in the original context can be achieved in the target context.

The conceptual PIET-T process model, on the basis of a systematic review, provides a set of criteria for evaluating transferability of health interventions. The model includes four domains to consider when taking an intervention into a new setting: population (how similar or different are the original and target populations?), intervention (how generalizable and relevant is the evidence to the new setting?), environment (what factors in the local or organizational setting might impact implementation?), and transfer (what communication and knowledge transfer is required to support adoption in the new setting and what is the involvement of local stakeholders?).^17^ Analyses of these factors can provide decision makers and researchers with the data to determine if or what additional research is needed, any necessary adaptations, further develop the intervention, and design implementation strategies for EBI transfer to the target context.

We emphasize the role of measures of implementation in the T transfer component of PIET-T because this step goes beyond communication and knowledge transfer and encompasses a wide range of factors that may influence the adoption and sustainability of the intervention in the new setting. Thus, in addition to the elements of T described in the PIET-T model (communication, knowledge transfer, strategies for adoption, evaluation, and sustainability), it is important to assess outcome measures for implementation such as reach, adoption, feasibility, and cost. Additionally, the transfer process may involve not only the intervention itself but also strategies for its implementation. For example, a diagnostic test may perform similarly in a HIC or LMIC setting, but its adoption and implementation within the health system may require very different strategies in distinct contexts. In the following section, we will apply the PIET-T model to two case studies as learning examples that involve implementation of tobacco use treatment guidelines and self sampling for HPV DNA in cervical cancer screening (Table 1).

**TABLE 1:**
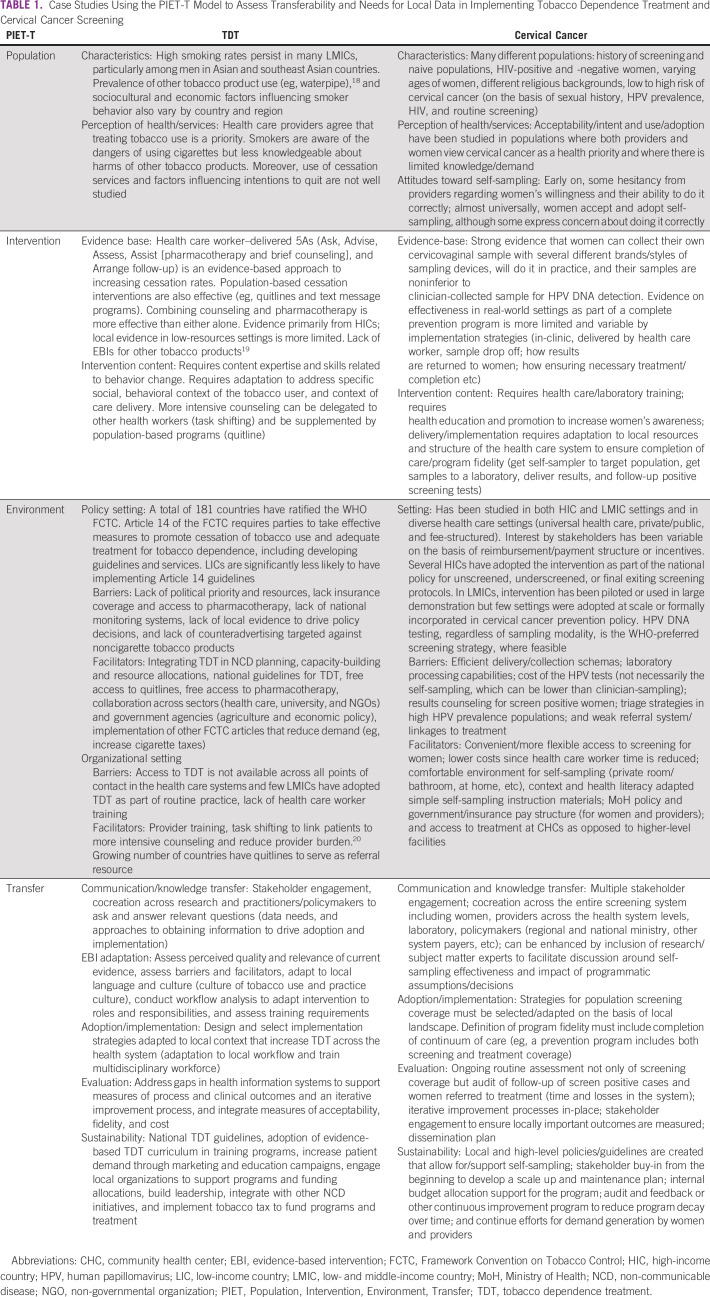
Case Studies Using the PIET-T Model to Assess Transferability and Needs for Local Data in Implementing Tobacco Dependence Treatment and Cervical Cancer Screening

## CASE EXAMPLES ASSESSING TRANSFERABILITY

### Case Study 1: Implementing Tobacco Use Treatment Guidelines in Vietnam

#### Intervention evidence.

There is an extensive literature showing that health care worker–delivered brief advice to quit, multisession counseling, and pharmacotherapy are effective and affordable approaches to increasing tobacco cessation compared with placebo.^21^ This literature is the basis for the international guidelines for promoting adequate treatment for tobacco dependence.^22^ Additional research, conducted primarily in HICs, provides evidence for effective strategies to increase adoption and implementation of these evidence-based tobacco cessation interventions in health care systems.

#### Local evidence.

In LMICs, local data on effective strategies for implementing tobacco use treatment in health care settings in LMICs are lacking and there is often a lack of local data demonstrating clinical outcomes that match those achieved in trials conducted in HICs. The lack of local evidence may create roadblocks to achieving equitable access to treatment.

#### Context.

The WHO has defined tobacco control policies and interventions, including tobacco cessation services as a best buy for reducing the burden of noncommunicable diseases, including cancer. This designation acknowledges the wealth of data supporting effective tobacco use treatment interventions. Yet, implementation has lagged, particularly in LMICs.^23^ The most frequently reported barriers include a lack of political will or prioritization among policymakers, a lack of health care system infrastructure (eg, treatment not integrated into primary care and health care worker training), and lack of funding, which is largely related to lack of political support for associated resource allocations.^24^ In the case of Vietnam, despite significant progress implementing the WHO Framework Convention on Tobacco Control policies and programs, there was no plan to implement treatment in community health centers (CHCs), the foundation of the primary care delivery system. Exploring this issue with policymakers indicated a skepticism about workforce capacity in CHCs to effectively treat tobacco dependence and a lack of resources to test that hypothesis.

#### Approach.

A partnership between local experts and both local and US investigators identified barriers and facilitators, and resulted in a plan that balanced policymakers' priorities to generate local evidence for the effectiveness of treatment delivered by trained providers and community health workers (does it work?) with the opportunity to simultaneously demonstrate the effectiveness of strategies, adapted to local context, to enhance implementation and guide scale up in CHCs nationally (how do we get it to work in varying contexts). A stakeholder-engaged formative assessment resulted in training and system changes (eg, decision support and referral system), adapted to local language, culture, health care provider workflows, and CHC infrastructure, which were feasible to incorporate into routine care and acceptable to end users. The partnership leveraged the existing public health system infrastructure, which includes a national village health worker program, to create a task-sharing model that linked CHCs with this community-based workforce, to facilitate implementation of evidence-based tobacco use treatment and smoking abstinence.

#### Conclusions.

There is extensive evidence for best practices in treating tobacco use as well as effective strategies to integrate treatment into routine practice. However, deferring to country partners' expertise and local knowledge is an important component of program adaptation, implementation, sustainability, and scale up. Policymakers may continue to require some degree of local data on treatment effectiveness to support decisions about resource allocation and scale up. It is possible to respond to these priorities in a timeline consistent with local needs for data by applying a range of rapid-cycle pragmatic designs to assess the quality of an adapted intervention, and the usefulness and acceptability of adapted strategies and patient outcomes. The findings from this approach can strengthen confidence that outcomes can be reproduced, thus, accelerating translation of tobacco use treatment evidence into public health policy and practice.

### Case Study 2: Self-Sampling for HPV DNA-Based Screening for Cervical Cancer

#### Intervention evidence.

There is an extant global literature showing that almost all women across diverse settings around the world say that HPV self-sampling is acceptable.^25-27^ In a meta-analysis, participants who took their own samples were twice as likely to accept HPV screening.^28^ Additionally, numerous studies have shown that women can properly collect their own samples for HPV-based cervical cancer screening and that HPV DNA can be detected in a way that is noninferior to HPV DNA detection on paired clinician-collected samples.^29,30^

#### Local evidence.

Despite evidence from a variety of settings, local leaders sometimes remain skeptical about whether a self-sampling protocol will be accepted and successful in their setting and may be reluctant to support it without local evidence. In some settings, pilot studies continue to be conducted to test whether local women will adopt self-sampling. It is not always clear what drives these calls for local evidence, but they appear to be driven by a belief that data on acceptability are needed from the specific setting and population. In fact, there are two questions related to local evidence that may arise as national or local leaders decide whether or how they should adopt the self-sampling strategy for cervical cancer screening: (1) Will women here do self-sampling? (2) Does self-sampling work here to detect HPV DNA in a manner noninferior to clinician-collected samples?

#### Context.

The WHO has called for the elimination of cervical cancer, and has laid out three pillars to achieve this goal: vaccination of 90% of girls by age 15 years; screen 70% of women by age 35 years and again by age 45 years; and treatment or management of 90% of women with cervical precancer or cancer. They have put forth guidance on what screening strategies to adopt but implementation challenges, including cost, have resulted in delayed or minimal scale up of HPV-based screening in many LMICs. Self-sampling for HPV represents a promising strategy for cervical cancer screening since it could reduce the time a woman spends at a health clinic (financial and care-taking barriers), require fewer human resources without the need for a speculum examination, and the test is very sensitive with a high negative predictive value.

#### Approach.

At the start, it is important to understand why key stakeholders believe that data in their exact context are needed despite an extensive and diverse literature to support the intervention. Is it a gap in the knowledge of the context under which the evidence was generated, a gap in translating this evidence to key decision makers, or a belief that their setting differs in some meaningful way that has not been researched or reported? Are political, institutional, or other interests driving the call for local evidence in a well-studied area? Understanding the why can help researchers and stakeholders to prioritize data needs and direct limited resources. This highlights a fundamental partnership: content matter experts and local context experts. Together, they can undergo a process of knowledge exchange to understand how the evidence base for HPV self-sampling relates to, or could operate in, the local setting to determine whether additional pilot testing is, in fact, needed. Related, this partnership could work to broaden the role of pilot or demonstration projects, to go beyond effectiveness questions to concurrently collect implementation data to guide efforts to bring cervical cancer screening programs to scale with the goal of sustainability.

#### Conclusions.

Because HPV self sampling has been used in so many different populations and settings, little additional evidence with regards to acceptability and efficacy should be needed to transfer to a new setting. However, understanding the local health system structure and constraints is important to selecting or adapting dissemination and implementation strategies to support self sampling. Additionally, existing evidence highlights how the intervention must be conducted within the context of a strong continuum of care that can ensure proper management and referral to higher-level care facilities. Thus, for integration of HPV self sampling, system-wide stakeholder engagement is essential to create scalable, faithful, and sustainable impacts on cancer control.

## LESSONS FROM THE TWO CASE STUDIES

These two case studies illustrate how researchers, policymakers, practitioners, and consumers may approach assessing or addressing the need for local evidence from different perspectives and with different priorities. Stakeholders' requests for local evidence may appear to researchers to be inconsistent with the existing evidence base. At the same time, some forms of local evidence, such as data to guide implementation or understand specific barriers, may be overlooked despite their importance. Researchers are concerned with applying rigorous methods to explain how local context influences the effectiveness of interventions and what strategies can improve adoption, implementation, and sustainable impact. Implementers may seek local evidence to also help with priority setting and to answer questions about whether results across target populations are equitable, and what systems are needed to monitor the effects of the program. As uses and expectations around local evidence may be different for different groups, aligning these priorities through multistakeholder engagement in which all parties participate in defining the questions and cocreating the solutions is critical.

In practice, implementation of cancer control interventions may occur with or without this local evidence base. There are already many efforts to implement cancer control interventions in LMICs, sometimes using innovative approaches to overcome constraints in resources or capacity. However, limited data are available to evaluate these implementation efforts. A systematic review of more than 10,000 articles published between 1998 and 2016 on a range of health interventions in LMICs found that < 5% captured information to inform implementation efforts, such as understanding contextual barriers and facilitators or documenting adaptations.^31^ Capturing and reporting data from real-world implementation efforts could help address calls for local evidence and build a robust evidence base for implementation of EBIs across diverse settings. Additionally, the process of implementation mapping provides a practical tool for developing and evaluating implementation strategies, including conducting a local needs assessment, identifying implementation outcomes, and planning for evaluation.^32^

## CONCLUSIONS AND CONSIDERATIONS FOR RESEARCHERS

Local evidence can be relevant at multiple points along the stages of implementation research, moving from the preimplementation phase, through the process of implementation, to the postimplementation phase.^33^ Theobald et al^16^ outlined the defining characteristics of implementation science in global health, including the importance of contextualization of an intervention, addressing challenges relevant to the community, using responsive methods, being demand driven, democratizing research through multistakeholder and multidisciplinary approaches, focusing on processes and outcomes, and being real-world and real-time focused. Peek et al^15^ also highlighted the importance of substantive ongoing participation by stakeholders to produce research that is relevant, which is a critical consideration when taking an intervention developed in a HIC setting and studying it in an LMIC.

Thus, in closing, we draw from the broader literature and our own experiences in global cancer control to offer some additional tools and best practices for researchers to consider:

- Engage stakeholders: Whole system stakeholders should be involved from the start in identifying priorities, framing research questions and participating in study design decisions to both build commitment and to ensure that relevant data needs are addressed. Stakeholder groups should include implementers as well as policymakers.
- Apply conceptual frameworks: Conceptual frameworks and theories, such as the Consolidated Framework for Implementation Research^34^; Reach, Effectiveness, Adoption, Implementation, Maintenance^35^; or the Exploration, Planning, Implementation, Sustainment framework,^36^ can help to define the context of intervention implementation at multiple levels and anticipate data needs, particularly related to barriers and facilitators. Frameworks and theories can also inform adaptations and design of strategies on the basis of local data.
- Use hybrid study designs when feasible: Study designs should consider and include process, context, and outcome measures aligned with stakeholder priorities. In situations where general evidence is limited in LMICs, hybrid designs allow for rigorous tests of both the intervention effectiveness and the implementation strategy and, at a minimum, should include studying contextual factors relevant to implementation, sustainability, and scale up.^37^
- Promote standardized context reporting: Publications often lack information about context. Standardized reporting of the context in which an intervention was tested would allow for greater understanding of the role of local factors and transferability. This could be developed in a manner similar to guidelines for reporting adaptations to interventions to support ongoing research.^38^ More comprehensive reporting will aid others in understanding how findings from one setting may or may not relate to other settings.
- Embed capacity building: To account for local context in the design, conduct, and interpretation of research studies, it is essential to have participation of skilled local researchers. In LMIC contexts where research capacity may be limited, contributing to training and capacity building is an important step for future research. Capacity building to inform implementation and build local evidence should be embedded in research activities to promote equity, identification of local priorities, and to strengthen stakeholder partnerships and roles.^39^

Local, contextual evidence can be important for both researchers and practitioners, and its absence may hinder translation of research and implementation efforts across different settings. However, it is essential for researchers, practitioners, and other stakeholders to be able to clearly articulate the type of data needed and why it is important. In particular, where resources are limited, evidence generation should be prioritized to address real needs and gaps in knowledge. Through researchers and practitioners working together from the start to identify data gaps relative to the local context, evidence generation can be focused where it is most critical and can support efficient implementation of EBIs across diverse contexts to support global cancer control.
